# Serum-Infliximab Trough Levels in 45 Children with Inflammatory Bowel Disease on  Maintenance Treatment

**DOI:** 10.3390/ijms18030575

**Published:** 2017-03-07

**Authors:** Helena Rolandsdotter, Per Marits, Ulf Sundin, Ann-Charlotte Wikström, Ulrika L. Fagerberg, Yigael Finkel, Michael Eberhardson

**Affiliations:** 1 Department of Gastroenterology, Sachs’ Children and Youth Hospital, SE-11883 Stockholm, Sweden; yigael.finkel@ki.se; 2 Department of Clinical Science and Education, Södersjukhuset, Karolinska Institute, SE-11883 Stockholm, Sweden; michael.eberhardson@ki.se; 3 Department of Clinical Immunology and Transfusion Medicine, Karolinska University Hospital, SE-17776 Stockholm, Sweden; per.marits@sll.se (P.M.); ulf.sundin.49@gmail.com (U.S.); ann-charlotte.wikstrom@sll.se (A.-C.W.); 4 Department of Medicine Solna, Karolinska Institute, SE-17776 Stockholm, Sweden; 5 Department of Women’s and Children’s Health, Karolinska Institute, SE-11776 Stockholm, Sweden; ulrika.fagerberg@regionvastmanland.se; 6 Center for Clinical Research and Department of Pediatrics, Västmanland Hospital Vasteras, SE-72189 Västerås, Sweden; 7 Department of Gastroenterology, Danderyds Hospital, SE-18288 Stockholm, Sweden

**Keywords:** inflammatory bowel disease, Crohn’s disease, ulcerative colitis, trough levels, antibodies toward infliximab

## Abstract

The role of trough serum infliximab (s-IFX) and antibodies toward IFX (ATI) during maintenance treatment remains unclear in children. The aim of the present study was to investigate trough s-IFX and ATI to identify any correlation with inflammatory activity and clinical response in a pediatric inflammatory bowel disease (IBD) cohort. We investigated the s-IFX trough levels in pediatric IBD patients (*n* = 45) on maintenance IFX treatment. Ninety-three blood samples were collected and demographics, C-reactive protein (CRP), erythrocyte sedimentation rate (ESR), and albumin were recorded. The mean s-IFX trough level was 5.2 µg/mL. The mean trough s-IFX level was significantly higher in the samples taken during remission (7.2 µg/mL) compared to active disease (4.5 µg/mL, *p* < 0.05). The trough s-IFX levels correlated with ESR, CRP, and albumin. S-IFX was undetectable in eight of the patients, all with positive ATI and active disease. Surprisingly, clinical and biochemical remission was observed at only 26 of the 93 visits. The correlation between dose variations and changes in trough s-IFX was not evident. In line with studies in adults, the s-IFX trough levels correlated with response to infliximab.

## 1. Introduction

Infliximab (IFX, Remicade^®^) is a chimeric monoclonal IgG_1_ antibody against tumor necrosis factor (TNF), a central cytokine in inflammatory bowel disease (IBD). This drug is effective in inducing and maintaining remission in Crohn’s disease (CD) and ulcerative colitis (UC), the two principal entities of IBD [1,2,3].

Baert et al. identified the potential immunogenicity of IFX in IBD patients, suggesting that patients may develop antibodies toward IFX (ATI), especially with episodic administration of the drug [4]. However, 6%–17% of the patients develop ATI even with scheduled treatment [5,6]. The association of ATI and infusion reactions is clear, and several studies demonstrate significantly lower trough serum IFX (s-IFX just before the next infusion) in patients with ATI, explained by increased elimination of IFX [4,7]. Low s-IFX and ATI formation have been associated with loss of response (LOR), but it has been difficult to establish any significant correlation between ATI and clinical parameters other than LOR as shown in a meta-analysis including 10 studies and 668 patients [4,8,9,10]. Treatment response seems to be more related to drug levels rather than to ATI, and a clear correlation has been found in several reports between trough s-IFX and clinical remission, C-reactive protein (CRP), and endoscopic improvement in adult patients [8,9,11,12,13].

The role of monitoring s-IFX and ATI, therapeutic drug monitoring (TDM), in the clinic is unclear, and the level of evidence is low given a limited number of studies with small cohorts, the use of retrospective designs, and different methodological approaches [10,14,15]. During induction of IFX, low trough levels have been suggested to support dose escalation in case of poor response [16,17,18]. In patients with loss of response, TDM may support dose escalation or switch to another drug [7,18,19]. A majority of the TDM studies have been performed in adult patients, and even though there are findings suggesting different pharmacodynamics and kinetics in children, very few studies have been undertaken to assess trough levels and ATI in pediatric cohorts [20,21,22,23]. The current study includes 45 children with CD or UC on maintenance IFX treatment. The study aims at correlating clinical activity and response to treatment with s-IFX levels and ATI in children.

## 2. Results

Forty-five children receiving IFX maintenance treatment were included and patients contributed with one sample for each visit with a total of 93 specimens; 15 patients contributed with one sample, 19 patients with two samples, four patients with three samples, and seven patients with four samples. The median age of this pediatric cohort was 16.0 years (range 7–18). CD was diagnosed in 32 patients (71%) and 13 patients (29%) had UC. In the anti-TNF-treated CD cohort, 27 patients (84%) had colonic or ileocolonic inflammation and 15 children (47%) also had involvement of upper gastrointestinal tract (mucosal inflammation found proximal to the ligamentum Treitz) at diagnosis. A proportion of 84% showed an inflammatory phenotype without stricturing or penetrating disease (B1). Only four patients (13%) had penetrating phenotype (B3) and seven children (22%) presented with perianal disease (P). Among the UC patients, 11/13 (85%) had extensive colitis or pancolitis (Table 1).

**Figure 1:**
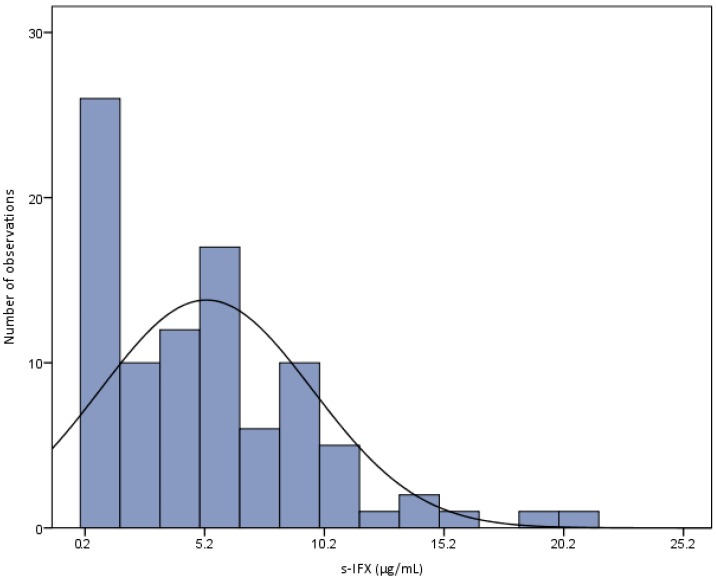
The distribution of serum infliximab (s-IFX) levels in 92 serum samples from 44 maintenance-treated pediatric IBD patients (one to four samples per patient) obtained immediately before the next scheduled infusion. Mean s-IFX trough level was 5.2 µg/mL (Range <0.2 to 21). One outlier of 40 µg/mL was excluded.

The duration of the IFX treatment ranged from 3 to 60 months, and the children had received a mean number of 13 IFX infusions (range 4–48). The mean dose of IFX ± standard deviation (SD) was 6.4 ± 1.7 mg/kg (median 6.2 mg/kg, range 3.44–10.5) with a mean interval of 44.8 ± 11.2 days. The mean s-IFX trough level was 5.2 µg/mL (median 4.5 µg/mL; range from <0.2 to 21), showing a right-shifted Gaussian distribution, as seen in Figure 1. One CD patient in remission with s-IFX 40 µg/mL was excluded from the analysis.

**Figure 2:**
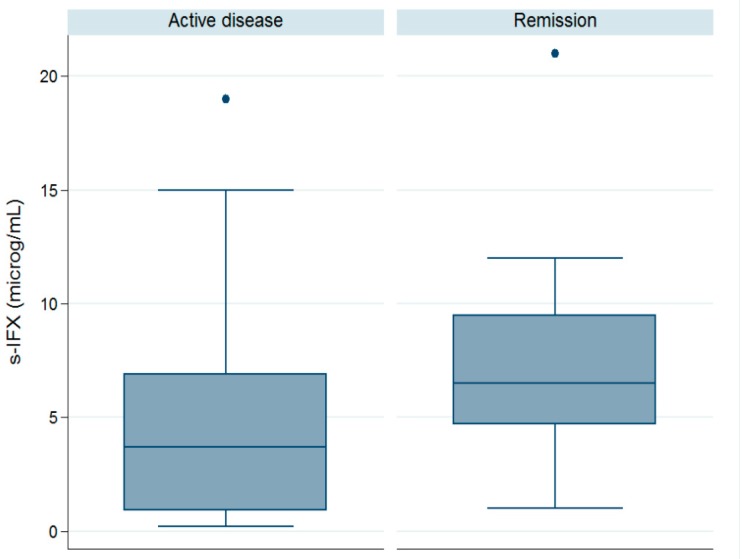
The mean trough s-IFX level was significantly higher in the samples taken during remission (7.2 µg/mL) as compared with s-IFX in active disease (4.5 µg/mL, *p* < 0.05). Clinical remission was assessed from activity scoring: PCDAI < 10 or PUCAI < 10, ESR < 10, and CRP < 5. One outlier of 40 µg/mL was excluded.

The assessment of disease activity was based on the validated scoring indices Pediatric CD Activity Index (PCDAI) and Pediatric UC Activity Index (PUCAI). The children were in clinical remission at 44 out of 93 visits (47%). With a stricter definition of remission using a combination of low clinical scoring and normalized C-Reactive Protein (CRP, mg/L) and Erytrocyte Sedimentation Rate (ESR, mm/h), the patients were in remission at 26 of the 93 test occasions (28%). Nine children were in remission at all visits, while 28 children were not in remission at any visit (10 of these non-remitters had only one visit). The clinical indices and biochemistry are summarized in Table 2. As shown in Figure 2, s-IFX was significantly higher in samples taken during remission (mean 7.2) as compared with sera collected during active disease (mean 4.5 µg/mL, *p* < 0.05). No significant difference was observed in dose-interval (days) between patients in active disease and those in remission (mean 43.0 days in active disease vs. mean 42.7 days in remission, *p* = 0.88) or in mean dose of IFX between the children in active disease (6.4 mg/kg) and those in remission (6.5 mg/kg, *p* = 0.76).

**Figure 3:**
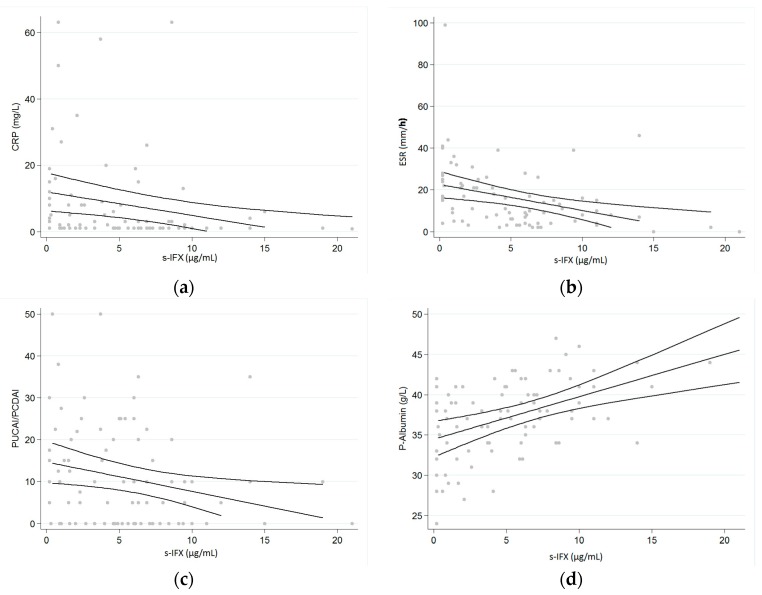
(**a**–**d**) Serum-IFX is correlated with the disease activity of the patients. S-IFX showed a negative correlation with: (**a**) CRP levels (*p* = 0.0084, *r*^2^ = 0.0491); (**b**) s-ESR (*p* = 0.0035, *r*^2^ = 0.1388) and (**c**) activity scoring PUCAI and PCDAI (*p* = 0.0259, *r*^2^ = 0.0687), and a positive correlation with: (**d**) s-Albumin (*p* = 0.0005, *r*^2^ = 0.2182). One outlier of 40 µg/mL was excluded.

The trough levels indicated a statistically significant correlation with clinical indices, as well as with CRP, ESR, and albumin levels, as illustrated in Figure 3a–d. No correlation was detected between trough levels and Fecal Calprotectin (FCP, mg/kg), and no difference was noted in s-IFX trough levels between CD and UC (not shown).

**Figure 4:**
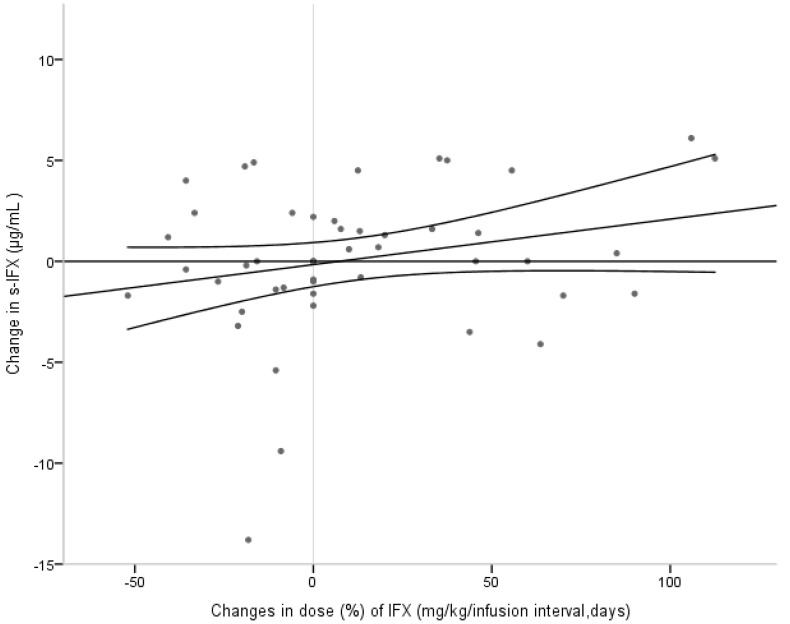
Change in dose (%) of IFX (mg/kg/dosing in days) did not show significant correlation with change in IFX trough levels in the 30 children who supplied two to four samples each (*p* = 0.58). The changes in s-IFX were based on samples obtained between approximately one to thirteen months apart.

Interestingly, intra-individual variations in dosing (mg/kg/infusion interval in days) did not show any clear correlation with changes in trough levels (µg/mL), as seen in Figure 4. Dose changes were not planned within the framework of the study, and were prescribed by the treating physician at his or her own discretion and not necessarily based on trough levels of s-IFX. The dose variations were also due to changes in weight. In the 30 patients who supplied two to four tests (total 48 samples) we identified 18/48 (38%) tests with decreased dose (mean −22%, SD ±13%), and in 11 of these 18 samples s-IFX decreased. Of these 18 samples, seven represented children in remission. At 22/48 (46%) test occasions, there was a dose increase (mean +44%, SD ±32%), and 15/22 tests showed subsequent increased s-IFX. Of these 22 samples, 17 tests represented patients with active disease.

In 12 samples from eight children (seven with CD and one with UC) collected at different sampling occasions, s-IFX trough levels were below detection and all of these samples were positive for ATI. None of the eight children were in remission at the time of the ATI positive samples. In six additional patients s-IFX was detectable, but below 1.0 µg/mL, giving a total of 14 patients with s-IFX of <1.0 µg/mL. All but one of these 14 patients showed active disease (CRP ≥ 5, ESR ≥ 10, and/or PCDAI ≥ 10 or PUCAI ≥ 10).

Of 14 patients with s-IFX of <1.0 µg/mL only two children had concomitant immunosuppressives during maintenance treatment. In the whole set of 93 s-IFX trough samples, 28 (30%) were collected at the time of concomitant immunosuppression. Mean trough IFX in these samples was 6.5 µg/L (0.2–21) compared with 4.8 µg/L (0.2–14) in samples from patients on monotherapy (*n* = 65, 70%) without reaching a significant difference between the two groups.

## 3. Discussion

The present study investigated s-IFX trough levels and ATI in a cohort of 45 pediatric IBD patients comprising IFX-treated children in the counties of Stockholm and Västmanland. S-IFX trough concentrations showed a significant correlation with clinical response and inflammatory activity. The intra-individual variations in the trough levels between visits were evident, and there was no clear correlation with dose changes, as seen in Figure 4. Low s-IFX trough levels were associated with the formation of ATI.

To our knowledge, there are only a few reports on IFX trough levels in children. In our study, we found a relatively high trough level (mean 5.2 µg/mL, median 4.5 µg/mL), even though within the proposed therapeutic interval 3–7 µg/mL for adults [18]. Hämäläinen et al. and Hoekman et al. both reported a median s-IFX of 3.5 µg/mL in a mixed pediatric UC and CD cohort, whereas Adedokun et al. reported a median s-IFX of 1.9 µg/mL at week 30 and 2.6 µg/mL at week 46 in a UC population receiving 5 mg/kg IFX q8w [22,24,25]. In the latter study, even a double dose of 10 mg/kg only gave a median trough of 2.9 µg/mL [24].

In our study, the children who responded to IFX and who were in clinical remission based on combined clinical index activity and biomarkers presented a significantly higher mean trough concentration compared to non-remitters (7.2 vs. 4.5 µg/mL). Previous reports have not shown consistency with regard to the correlation between serum IFX levels and clinical activity indices/biomarkers [22,24,25]. In adults, the correlation between pre-infusion s-IFX and clinical response is well established [11,26].

Surprisingly, the children were in clinical remission, defined as a reduction in the clinical activity index as well as normalization of CRP/ESR, only at 26 of the 93 visits during maintenance treatment. This finding should be interpreted with some caution in the light of the restrictive definition of remission in this study, including clinical indices as well as normalization of biomarkers. Previous reports on IFX and clinical response in children have been divergent, and some studies suggest high rates of LOR up to 50% in children [20,21,27,28,29]. The finding of only 28% remission rate based on the 93 recorded visits was surprising, especially in light of the relatively high mean trough level of 5.2 µg/mL in this report. A sub-study of the pivotal Crohn trial with IFX (the ACCENT I trial) revealed median week 14 trough levels of 4.0 µg/mL in patients with sustained response to IFX 5 mg/kg and 1.9 µg/mL in patients without sustained response [30].

We could not detect any clear impact of dose changes on the s-IFX levels. In this study, the s-IFX is a mixture of tests taken in the clinic by the treating physician, as well as tests obtained within the current study. Therefore, not every decision to change dosing has been based on s-IFX levels. Moreover, since the dosing also depends on weight and infusion interval, the variation may not be a result of active dosing decisions.

ATI was found in all patients with undetectable s-IFX, and all these patients had active disease. This observation may suggest that ATI plays an important role in children with low trough levels and incomplete response to the treatment. Yet, a major limitation in the enzyme-linked immuno sorbent assay (ELISA) analysis of ATI is the inability to detect antibodies in the presence of IFX residue, which makes it difficult to speculate about the role of ATI in children exhibiting low but detectable trough levels. Vande Casteele et al. investigated the relationship between IFX concentrations, ATI, and disease activity in 1487 IFX trough serum samples from 483 adult CD patients [26]. Their method allowed for the analysis of ATI in the presence of IFX, showing that ATI even at low as well as therapeutic concentrations of IFX increased the probability of active disease. It is conceivable that the same condition prevails in the pediatric population. The combination therapy with immunosuppressives is thought to reduce the frequency of ATI. We found a numerical trend toward a higher s-IFX concentration in children on azathioprine, but without reaching statistical significance, probably due to the small number [4,10]. Children were on concomitant immunosuppressant only at 30% of the visits during maintenance treatment. This could probably reflect the fear for hepatosplenic T-cell lymphoma in young male patients [31].

Whether TDM is a valuable tool to maintain remission in patients with IBD is not clear. In a randomized controlled study including 263 adult IBD patients dosing was optimized for IFX trough levels 3–7 µg/mL at the start and the patients were then randomly assigned to either continued TDM-based or clinically based dosing [32]. The study showed no superiority of concentration-based dosing after one year with regard to remission rates. However, TDM-based dosing was associated with fewer flares during the course of treatment. Furthermore, a retrospective study examining the use of proactive TDM in 48 adult patients compared to 78 patients with standard care demonstrated a higher probability of remaining on IFX in patients with s-IFX trough levels >5 µg/mL [33]. In a study by Minar et al., TDM was evaluated in IFX-treated children with CD who experienced LOR. The authors found that ESRs at the previous infusion were significantly associated with IFX concentrations [23]. The current report is of observational and retrospective nature. Interventions were not implemented within the study and therefore conclusions with regard to the role of TDM-based IFX dosing are beyond the scope of this report. Nevertheless, in this cohort with children not subjected to active TDM, only one third was in clinical and biochemical remission.

## 4. Materials and Methods

### 4.1. Patients

Between September 2013 and May 2015 all identified pediatric IBD patients (*n* = 45, age 7–18 years) on IFX maintenance treatment in the counties of Stockholm and Västmanland were enrolled in this retrospective cohort-study. The inclusion criterion was current maintenance IFX treatment after at least three induction doses for the indication of either CD or UC. Serum samples of 2 mL were obtained before scheduled IFX infusions and analyzed for s-IFX (trough level) using an in-house-developed ELISA. In the study, at least one sample was obtained from each identified child on maintenance IFX and moreover, additional trough s-IFX already taken at the discretion of the treating physician outside this study was also included in the analysis. Therefore, the cumulative number of IFX trough samples for each patient differed (see result section). Any dose adjustment was done at the discretion of the treating physician. The clinical information collected from the medical charts included age at IBD diagnosis, type of IBD diagnosis, Paris classification, age at inclusion in the study, duration of IFX treatment, current dose correlated to weight, infusion interval in days, and total number of IFX infusions. Concomitant treatment of immune modulators (azathioprine) was registered. Comorbidity with primary sclerosing cholangitis (PSC), diabetes mellitus, vasculitis, celiac disease, and arthritis were also documented (see Table 1 for patient characteristics).

The sera were separated and handled according to standard operative procedures until analysis. Quantification of serum levels of IFX was performed at the Department of Clinical Immunology, Karolinska University Hospital, Stockholm with ELISA assay used in clinical routine in the Stockholm and Västmanland counties. ATIs were analyzed in samples with undetectable (<0.2 µg/mL) trough levels of IFX. CRP (mg/L), ESR (mm), and albumin (g/L) were registered at every infusion. FCP (mg/kg) was collected in 34/45 patients. PUCAI and PCDAI were calculated at the time of IFX infusion, except for approximately 20% which were calculated retrospectively based on charts. CD patients were considered in remission when the PCDAI was <10, CRP < 5, and ESR < 10; UC patients were considered in remission if the PUCAI was <10, CRP < 5, and ESR < 10 [34,35]. Three CRP and 11 ESR samples were missing, and in the case of missing values the assessment of remission or active disease was based on available biomarkers together with the clinical activity index. Dose changes were calculated by dividing dose (mg) with weight (kg) and infusion interval (days).

### 4.2. IFX ELISA

The trough level concentration in serum samples was measured with an in-house developed and validated ELISA methodology used in clinical routine, which has been described previously [11]. Briefly, the microtiter plates (Nunc Maxisorp F 96, Thermo-Fisher Scientific, Roskilde, Denmark) were coated with 100 ng/mL, 50 µL/well of recombinant human TNF-α (R&D Systems, Minneapolis, MN, USA) in 0.05 M sodium carbonate buffer pH 9.6. The plates were put on a shaker at room temperature (RT) for 2 h and incubated overnight at +4 °C. The plates were washed three times in phosphate buffered saline (PBS) plus 0.05% pH Tween 20 and blocked with PBS + 1% bovine serum albumin (BSA) (Sigma, St. Louis, MO, USA) and 0.05% Tween 20 (blocking buffer) for 1 h at RT. After an additional wash, standard dilutions (0.40–100 ng/mL) of IFX (Schering Plough, Kenilworth, NJ, USA) were added to the plate together with defined IFX-spiked sera (internal controls) and patient samples, diluted 1/500 in blocking buffer, all in duplicates. The plates were incubated on a shaker at RT for 1 h and washed four times followed by the addition of alkaline phosphatase (ALP)-conjugated goat anti-human IgG (Fc-specific) (Sigma) diluted 1/10,000 in a blocking buffer. After incubation for an additional hour on a shaker, the plates were washed four times. Substrate (*p*-nitrophenyl-phosphate, 5 mg/mL in 1 M diethanolamine with 0.5 mM Mg, pH 9.8) was added and color development was monitored at 405 nm. The concentration of samples and controls was calculated from the standard curve: lower and upper limits of quantification were 0.2 µg/mL and 50 µg/mL, respectively (compensated for serum dilution 1/500).

### 4.3. Inhibition ELISA for ATI Detection

ATI were analyzed with an in-house developed ELISA based on the inhibition of binding of labeled IFX to TNF-α coated to the ELISA plate, as previously described [11]. ALP was coupled to IFX using the Lightning-Link kit (Innova Biosciences Ltd., Cambridge, UK). The plates were coated as described above and washed three times in PBS plus 0.05% pH, Tween20 and incubated with blocking buffer for 1 h at RT. A standard consisting of goat anti-human IgG (see above) at a final concentration of 1 µg/mL and diluted serum samples were incubated with ALP-conjugated IFX for 1 h at RT. After an additional wash of the TNF-coated plate, aliquots of standard solutions and samples in duplicates were transferred to the plate, which were then incubated on a shaker for 1 h at RT. After additional washes, substrate (see above) was added and color development at 405 nm was monitored. The results were transformed to percentage inhibition by normalization of the samples’ OD to that of the standard (100% inhibition) using the formula (OD blank − OD sample)/(OD blank − OD standard) × 100. The lower limit of detection was set to the value plus two standard deviations obtained from measurements of normal control sera. ATI could only be detected in the absence of the drug because of interference of IFX in the assay. Thus, ATI detection was limited to patients with undetectable s-IFX (<0.2 µg/mL).

### 4.4. Statistical Analysis

Correlations between clinical parameters, routine chemistry, and s-IFX were assessed with linear regression with cluster-robust standard error to test univariate associations between clinical variables and s-IFX (dependent). Other intergroup comparisons were performed with two-tailed *t*-test. The correlation between changes in dose and concomitant changes in trough levels was assessed with simple linear regression (Pearson). Statistical analysis was performed with IBM SPSS Statistics 23 Data Editor^®^ software, and Stata 13.1. Significance was set at *p* < 0.05. In all statistical analyses one outlier (IFX trough level 40 µg/mL) was excluded.

### 4.5. Ethical Approval

The study was done in accordance with the Helsinki II Declaration and approved by the local Ethics Committee in Stockholm (Approval No: 2012/378-31/3, 6 December 2012). Informed written consent from all patients over 15 years and parents for children aged less than 15 years was obtained before any study-related procedure.

## 5. Conclusions

In conclusion, this retrospective study of s-IFX and ATI formation during maintenance treatment in children with IBD demonstrates a correlation between clinical as well as biological response to the therapy and trough s-IFX. However, the number of children in the present study is too small to allow anything but tentative conclusions regarding dosing and therapeutic interval. Therefore, the results warrant larger prospective studies to determine optimal use of IFX in children with UC and CD.

## Figures and Tables

**Table 1:** Background characteristics of the patient cohort. IBD, inflammatory bowel disease.

Patient Characteristics and Disease Classification		Number (%)
Total number of patients		45
Male sex, number (%)		29 (64%)
Age at inclusion, median (range), years		16 (7–18)
Age at IBD diagnosis, median (range), years		12.0 (2–17)
IBD onset (age)	≤10 years	31%
	11–15 years	58%
	16–18 years	11%
Immunosuppression (Azathioprine) at any time during the study		29/45 (64%)
Crohn’s disease *n* = 32 (71%), Paris classification at diagnosis		
L1 (distal 1/3 of ileum + caecum)		5 (16%)
L2 (colonic)		12 (37%)
L3 (ileocolonic)		15 (47%)
L4a (upper disease proximal Treitz)		15 (47%)
L4b (upper disease distal Treitz)		2 (6%)
B1 (non-stricturing/non-penetrating)		27 (84%)
B2 (stricturing)		1 (3%)
B3 (penetrating)		4 (13%)
B2B3 (stricturing/penetrating)		0
P (perianal disease)		7 (22%)
G0 (no growth delay)		29 (91%)
G1 (growth delay)		3 (9%)
Ulcerative colitis *n* = 13 (29%), Paris classification at diagnosis		
E1 ulcerative proctitis		0
E2 left-sided ulcerative colitis		2 (15%)
E3 extensive colitis		4 (31%)
E4 pancolitis		7 (54%)

**Table 2:** Disease activity parameters at time of sampling. PCDAI, Pediatric Crohn’s disease Activity Index; PUCAI, Pediatric Ulcerative Colitis Activity Index; CRP, C-reactive protein; ESR, erythrocyte sedimentation rate.

Disease Activity Parameters		Numbers (%)
PCDAI	<10 (remission)	34/93 (37%)
	≥10	31/93 (33%)
PUCAI	<10 (remission)	10/93 (11%)
	≥10	18/93 (19%)
CRP (mg/L)	Median (range)	2.0 (1–63)
	Mean	8.3
ESR (mm/h)	Median (range)	38 (2–99)
	Mean	17
Albumin (g/L)	Median (range)	38 (27–44)
	Mean	37
F-Calprotectin (mg/kg)	Median (range)	884 (15–9068)
	Mean	150
**Comorbidities**		
Primary sclerosing cholangitis		3
Celiac disease		1
Arthralgia/arthritis		4
Vasculitis		1
Diabetes mellitus		0

## Abbreviations

ALP	Alkaline phosphatase
ATI	Antibodies toward infliximab
CD	Crohn’s disease
CRP	C-reactive protein
ESR	Erytrocyte sedimentation rate
LOR	Loss of response
PBS	Phosphate buffered saline
PCDAI	Pediatric CD activity index
PSC	Primary sclerosing cholangitis
RT	Room temperature
TDM	Therapeutic drug monitoring
UC	Ulcerative colitis
PUCAI	Pediatric UC activity index
